# Reinforcement Learning: A Paradigm Shift in Personalized Blood Glucose Management for Diabetes

**DOI:** 10.3390/biomedicines12092143

**Published:** 2024-09-21

**Authors:** Lehel Dénes-Fazakas, László Szilágyi, Levente Kovács, Andrea De Gaetano, György Eigner

**Affiliations:** 1 Physiological Controls Research Center, University Research and Innovation Center, Obuda University, 1034 Budapest, Hungary; denes-fazakas.lehel@uni-obuda.hu (L.D.-F.); szilagyi.laszlo@uni-obuda.hu (L.S.); kovacs@uni-obuda.hu (L.K.); andrea.degaetano@biomatematica.it (A.D.G.); 2 Biomatics and Applied Artificial Intelligence Institute, John von Neumann Faculty of Informatics, Obuda University, 1034 Budapest, Hungary; 3 Doctoral School of Applied Informatics and Applied Mathematics, Obuda University, 1034 Budapest, Hungary; 4 Computational Intelligence Research Group, Sapientia Hungarian University of Transylvania, 540485 Tîrgu Mureș, Romania; 5 CNR-IASI Institute for Systems Analysis and Computer Science, National Research Council of Italy, 00185 Rome, Italy; 6 CNR-IRIB Institute of Biomedical Research and Innovation, National Research Council of Italy, 90146 Palermo, Italy

**Keywords:** blood glucose levels, diabetes, reinforcement learning, artificial intelligence, glucose control, personalized management, dynamic strategies, patient profiles, closed-loop insulin delivery systems, predictive models, monitoring

## Abstract

**Background/Objectives:** Managing blood glucose levels effectively remains a significant challenge for individuals with diabetes. Traditional methods often lack the flexibility needed for personalized care. This study explores the potential of reinforcement learning-based approaches, which mimic human learning and adapt strategies through ongoing interactions, in creating dynamic and personalized blood glucose management plans. **Methods:** We developed a mathematical model specifically for patients with type IVP diabetes, validated with data from 10 patients and 17 key parameters. The model includes continuous glucose monitoring (CGM) noise and random carbohydrate intake to simulate real-life conditions. A closed-loop system was designed to enable the application of reinforcement learning algorithms. **Results:** By implementing a Policy Optimization (PPO) branch, we achieved an average Time in Range (TIR) metric of 73%, indicating improved blood glucose control. **Conclusions:** This study presents a personalized insulin therapy solution using reinforcement learning. Our closed-loop model offers a promising approach for improving blood glucose regulation, with potential applications in personalized diabetes management.

## 1. Introduction

Diabetes mellitus (DM) is a persistent and currently untreatable metabolic disease resulting from either a complete lack of insulin (Type 1 Diabetes Mellitus, T1DM) or a partial lack of and/or an insufficient effect of insulin (Type 2 Diabetes Mellitus, T2DM). The exact pathophysiology is still unclear; TDM1 is believed to be the result of a cascade of autoimmune reactions that destroy the insulin-producing β cells in the Langerhans islets in the pancreas [1]. This manuscript focuses on managing blood sugar levels under the circumstances of T1DM using reinforcement learning methods of artificial intelligence techniques for external insulin administration.

T1DM typically develops rapidly and mostly affects children and adolescents, possibly genetically prone individuals. However, lifestyle and external circumstances an also accelerate the manifestation of the condition [2,3].

The fundamental molecular role of insulin is the insulin-stimulated entry of glucose into insulin-sensitive tissues (mainly muscle and adipose tissue) and the inslulin-mediated suppression of excessive glucose production (gluconeogenesys, glycogenolysis) mainly in the liver and the kidneys [4].

Patients with TDM1 need external insulin throughout their life to maintain their glycemia [5]. Without external insulin administration, these patients cannot survive due to the energy breakdown of their body’s household [6]. High-quality robust and personalized blood glucose management is of significant importance for patients with T1DM to maintain normal glycemia levels and to decrease the expression of side effects. In recent years, it was proven that semi-automated insulin administration in the case of T1DM is able to provide satisfactory glycemia levels over the years, and it is able to reduce the risk of developing serious side effects [7,8]. However, there is a strong need for further development in the area of control algorithm candidates that can be employed for insulin administration. Such solutions need to be robust at the beginning of usage but need to have self-tuning capabilities to adapt to the patient needs over time to provide personalized treatment and insulin administration [9].

Usually, semi-automatic glycemic control operates according to the artificial pancreas (AP) concept. An AP system consists of three components: a Continuous Glycemia Monitoring System (CGMS) to measure the glycemia, an insulin pump as an actuator to inject insulin, and an (advanced) control algorithm [9,10,11,12].

Control algorithms in artificial pancreas systems play a crucial role in determining insulin dosing based on real-time glucose data [13,14,15,16]. These algorithms continuously analyze glucose sensor readings and use mathematical models to predict future glucose levels. The primary goal is to maintain blood sugar levels within a target range while minimizing the risk of hypoglycemia (low blood sugar) and hyperglycemia (high blood sugar).

Various types of control algorithms have been developed and investigated over the two decades of AP developments, including for example proportional–integral–derivative (PID) controllers, model predictive controllers (MPCs), and fuzzy logic controllers. Each algorithm has its strengths and limitations, and researchers continue to refine and optimize them for better performance and safety [17,18,19,20,21,22].

The choice of control algorithm depends on factors such as the individual’s insulin sensitivity, meal intake, physical activity, and overnight glucose patterns. Additionally, advancements in machine learning and artificial intelligence have led to the development of more adaptive and personalized algorithms that can adjust insulin dosing based on individual variability and changing circumstances [23,24].

Current methods of blood glucose management in type 1 diabetes rely on regular self-monitoring of blood glucose levels and insulin administration. However, these methods require significant dedication from patients. As a result, there is increasing interest in utilizing machine learning techniques, particularly reinforcement learning (RL) [25,26,27].

Reinforcement learning (RL) belongs to the advanced machine learning methods, where an agent-based algorithm (so-called neural network-based model) learns the appropriate policy for acting upon training by receiving feedback in the form of rewards or penalties. Its effectiveness has been showcased in diverse fields such as game playing [28], robotics [29], and healthcare [30]. In the context of blood glucose management, RL can be utilized to formulate a personalized strategy for adjusting insulin dosages based on the analysis of both present and past blood glucose readings, injected insulin, and other possible features [31].

In this paper, we present our reinforcement learning-based solution for blood glucose control, which uses a PPO agent to control blood glucose levels. The experiments were performed using an IVP mathematical model. From this mathematical model, we created a closed-loop simulator that corresponds to a reinforcement learning environment. For this mathematical model, we had 17 parameter sets from 10 patients.

## 2. Related Works

Ref. [32] conducted a comprehensive study on the application of offline RL algorithms for glucose control, particularly focusing on Batch-Constrained Q-learning (BCQ) and Conservative Q-Learning (CQL). Offline RL is advantageous because it allows for training models on pre-existing datasets, thereby minimizing risks associated with real-time patient interactions during the learning process. The study reported a significant improvement in TIR, increasing from 61.6% to 65.3%, a result that is both statistically significant and clinically relevant. The research also emphasized safety using CVGA to confirm that the majority of glucose predictions fell within Zone A, demonstrating high clinical accuracy. This work is critical, as it paves the way for the safer deployment of RL-based glucose control in real-world settings, where patient safety is paramount.

Ref. [33] explored the use of the Soft Actor-Critic (SAC) algorithm, which is a model-free RL approach known for its ability to handle continuous action spaces effectively. The SAC was applied to optimize insulin dosing in a closed-loop system designed for type 1 diabetes management. The algorithm was specifically tailored to dynamically adjust insulin delivery in response to varying blood glucose levels, addressing the inherent variability in diabetic patient responses. The study achieved an impressive TIR exceeding 75%, which is a significant improvement over traditional methods. Furthermore, the CVGA results indicated that most glucose predictions were within the safe Zones A and B, confirming the algorithm’s reliability and safety. This research is particularly notable for demonstrating the feasibility of using advanced RL algorithms in real-time clinical applications.

Ref. [34] introduced a multi-step deep RL strategy designed to tackle the complexities of glucose regulation, particularly the delayed effects of insulin and meal intake on blood glucose levels. The study utilized advanced RL techniques such as Dueling DQN and Prioritized Experience Replay (PER), which enhance the algorithm’s ability to learn from important past experiences while stabilizing learning in the presence of delayed rewards. The results were remarkable, with a TIR of 85.62%, representing a 28.7% improvement over the baseline methods. The CVGA analysis further validated the model’s safety, with more than 90% of predictions falling within Zones A and B. This study highlights the importance of considering temporal dependencies in glucose control and showcases the potential of multi-step learning in addressing these challenges.

Ref. [35] focused on evaluating various extensions of the Deep Q-Network (DQN) algorithm for blood glucose control in in silico models of type 1 diabetes patients. The study compared different DQN variants, including Double DQN and NoisyNet-DQN, to determine which configurations were most effective in managing blood glucose levels. The research showed that these advanced DQN algorithms could significantly improve the TIR, reaching 80%, which is a substantial enhancement over traditional approaches. The CVGA analysis confirmed the safety of the control decisions, with most predictions within Zones A and B. This study is particularly important for its in-depth analysis of different DQN architectures and their implications for safe and effective glucose control.

## 3. Materials and Methods

### 3.1. Reinforcement Learning

Reinforcement learning is a flexible framework known for its ability to adjust to complex process demands, functioning through semi-supervised learning. In this study, we applied the actor-critic architecture [36]. The objective of this model was to identify the sequence of actions that maximizes the reward value by the combined actions of the actor and the critic (both are neural networks). Embodied within the actor-critic models is a neural duality, which is realized as two distinct neural networks: the first one is the value network aiming to quantify the intrinsic quality of states via a value function. The second one is the policy network that sets probabilistic values for actions (vector or scalar) in order to facilitate the comprehensive objective of maximizing rewards [36]. In this study, we applied the Proximal Policy Optimization (PPO) paradigm [37]—below, we explain why we chose PPO by presenting our preliminary results. The PPO is an evolution of the Stable Baselines3 library [38]. Policy gradient methods such as TRPO [39], GAE [40], and A2C/A3C [41,42] have recently come to attention. We worked within the OpenAI gym [43], which is an environment for the development of reinforcement learning situations.

### 3.2. Preliminary Results

In this section, we summarize the progress made so far through three sub-section, as we have published three articles on the subject. This manuscript draws on the conclusions of these studies and implements them together to achieve better results for both the TIR and CVGA metrics achieved in these articles. We describe the important conclusions of these manuscripts, but the full network we used in this research is described in the later Section 3.6.

#### 3.2.1. Testing Multiple Agents and Varying Simulation Lengths

In the previous study [44], the feature vector was calculated using blood glucose measurements and insulin administrations over one hour in the past. Since the measurement interval of the CGM sensor was 5 min, 12 samples of blood glucose were used.

During feature extraction, 11 features were extracted from the blood glucose data, and 11 features were also extracted from the insulin data. From the blood glucose data, we calculated 11 point deviations from the existing 12 measured points. Then, we also used the same method, only in the second case, we divided by five, since the sensor measures at every 5 min. We also extracted 11 features from the insulin data. This was also a pointwise variation. Also, there was a calculation of the difference between two endpoints for the insulin characteristics.

(1) ΔG(i)=G(i+1)−G(i),i∈[0,11]

(2) $$\frac{\Delta G(i)}{\Delta t}=\left(G\left(i+1\right)-G\left(i\right)\right)/5,\;i\in \left[0,11\right]$$

(3) Δu(i)=u(i+1)−u(i),i∈[0,11]

(4) Δu=u(11)−u(0)

In that first study, we examined several types of agents, including both discrete and continuous action space agents. The algorithms we investigated were the following:

- PPO [37];
- SAC [45];
- DDPG [46];
- DQN [47];
- A2C [48];
- TD3 [49].

We implemented a closed-loop system that included a virtual patient acting as the environment [50]. As the controller, our system included a neural network-based agent: this agent sends the insulin data to be “injected” into the environment and thus modify the environment; the environment, in turn, returns a blood glucose value based on the injected insulin, on set patient parameters, and on a given carbohydrate intake. The action space spanned insulin administration rates from 0 to 18 U/h.

We examined several types of reward functions for the optimization of the controller: the tests showed that a two-step reward function provided the best performance for our problem. In the first step, we calculated a temporary reward, and then in the second step, we averaged the temporary rewards. The tests were evaluated using the Time in Range (TIR) [51] and Continuous Variability Grid Analysis (CVGA) metrics [52]. Since the PPO algorithm yielded the best performance in the first tests, we then continued to work with this algorithm for the one-day test analysis. For the one-day test, we set up three different test cases, which are introduced here in order to provide a full overview of the fundaments of the current research results.

#### Preliminary Results Scenario I: 1 Day of Training, 1 Day of Testing

In our initial trial, we conducted a one-day simulation for instructional purposes, followed by a one-day assessment phase. Among the patients, five exhibited extreme glucose level fluctuations that led us to terminate the testing protocol. For the remaining patients, no remarkable deviations were observed in their glycemia time course. The average Time in Range (TIR) recorded was 77.55%, which is a satisfactory outcome considering the broader patient cohort; see Table 2 from [44]. The distribution pattern of blood glucose (BG) levels across all patients also aligned well with the TIR criterion.

By evaluating the results through the Control Variability Grid Analysis (CVGA) metric, we identified Zone B control actions for a total of seven patients. Notably, patient number 7 showcased the most commendable performance, maintaining a flawless 100% adherence within the target range; see Table 2 from [44].

#### Preliminary Results Scenario II: 1 Day of  Training, 10 Days of  Testing

For the next evaluation, we engaged the agent in a one-day training period followed by a comprehensive ten-day simulation period. The objective was to test the agent’s capacity to extrapolate from a single day of training towards an extended simulation period. During this trial, we encountered four instances of extreme blood glucose (BG) levels. In contrast to the previous trial, shifts in the agent’s performance out of the middle range were discernible, resulting in higher blood glucose (BG) levels.

According to the CVGA diagram in Table 3 from [44], only two instances were felt in Zone B. Considering the Time in Range (TIR) metrics, the average was 72%. Notably, patient 10 emerged as the standout performer in terms of the results. An intriguing scenario emerged with patient 13, wherein the agent managed to steer the patient away from extreme conditions while leaning towards higher BG levels within the non-extreme zone.

The 180–250 BG range was occupied on average 17% of the time, which is well within the stipulated acceptable threshold (25%). This alignment with the TIR metric confirms its consistency. However, for patient 13, the allowed percentage was exceeded, raising questions on the general applicability of the agent.

#### Preliminary Results Scenario III: 10 Days of  Learning, 1 Day of  Testing

For our third evaluation, we inverted the conditions of the second test: the agent underwent a ten-day training phase, which was followed by a condensed one-day testing period. The aim was to ascertain whether the agent’s ability to generalize from extensive training could be translated into shorter testing scenarios.

The outcomes of this test do not support the agent’s capacity to formulate control strategies for individual patients that effectively avert extreme zones: the Time in Range (TIR) metrics failed to align with the predefined criteria, as the blood glucose (BG) levels showed shifts towards the higher range.

These results suggest that we may have a good result if we use the same simulation duration for both training and testing. In this test, no CGM noise was used in the patients.

#### 3.2.2. Testing with Different Reward Functions

In our previous study [53], we investigated which reward functions would give the best performance in terms of blood glucose regulation. For the first time, we examined continuous functions as well. One of the main challenges here was that we used an extended mathematical model, namely, the virtual patient model was completed by a stochastic sensor noise (continuous glucose monitoring sensor—also known as CGMS). We applied the model output: the noisy blood glucose level in the reward functions. Because of this, the deviations of the different reward functions were much larger, which made training extremely difficult, as violations of the termination criterion boundaries occurred more often due to the deviations. On the other hand, this procedure increased the viability of the methodology, since in practice, the CGM signal is available as input for control actions.

The simulation time for both training and testing was one day. During training, carbohydrate inputs were randomly generated during exercise, and carbohydrate intake was randomly generated according to the Section 3.4. Thirteen possible test days were defined with different carbohydrate intake patterns. Ten test days had randomly generated carbohydrate intake, while three test days had predefined carbohydrate intake: (i) one had no carbohydrate intake, (ii) one had 12 g of carbohydrate intake every hour, (iii) one had an additional 5 g of carbohydrate at 12 h in addition to the carbohydrate amount generated by the meal function as described in Section 3.4. The control agent we used was the PPO algorithm, since this provided the best performance in our first tests according to Section 3.2.1. The architecture was not complex: both the actor and critic networks were outgoing networks. However, they were structurally identical and contained two hidden layers with a Relu activation functions in the hidden layer. Moreover, the observation space was changed, as there were now only two elements for the observation of blood glucose and insulin concentration at the previous time point.

**Figure 1:**
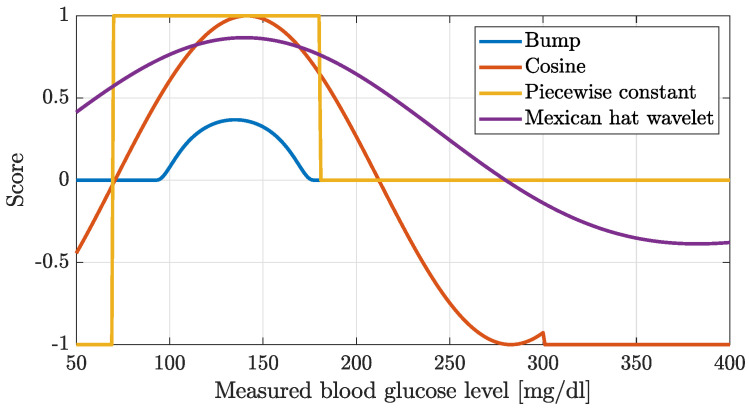
Investigated reward functions.

We tried four different reward functions shown in Figure 1, one of which was not continuous. All functions were maximized around the 120 mg/dL blood glucose level. We measured the performances of the reward functions by applying concrete termination criterion during training (and stopping the simulation when it was violated, namely, when the blood glucose level felt outside the given ranges) and by not terminating the simulation at all. We applied CVGA and the TIR for performance measurements as metrics. Furthermore, we also calculated the mean squared error of the blood glucose level that occurred during the test. This error was calculated both for 90 mg/dL and 150 mg/dL. In the following, we introduce the tested reward functions.

**Bump function [54]**:

(5) $$score\left(i\right)=\left\{\begin{array}{cc} \exp\left(\frac{-1}{1-{\left(\left(CGM-90\right)/45-1\right)}^{2}}\right) & 90<CGM<180\\ 0 & \mathrm{otherwise} \end{array}\right.$$

**Piecewise constant function**:

(6) $$score\left(i\right)=\left\{\begin{array}{cc} -1 & CGM<70\\ 1 & 70\leq CGM\leq 180\\ 0 & 180<CGM \end{array}\right.,$$

(7) $$\begin{array}{c} R=\frac{1}{n}\sum_{i=1}^{n}score\left(i\right), \end{array}$$

where *R* is the final reward, and *n* is the simulation length.

**Cosine function**:

(8) $$score\left(i\right)=\left\{\begin{array}{cc} -\cos\left(\frac{CGM}{45}\right) & 0<CGM<300\\ -1 & otherwise \end{array}\right.,$$

**Mexican hat wavelet [55]**:

(9) $$\begin{array}{c} score\left(i\right)=\frac{2}{\sqrt{3}\cdot \pi^{\frac{1}{4}}}\cdot (1-(\frac{CGM-140}{140}{)}^{2})\\ \cdot \exp\left(-(\frac{1}{2}\cdot (\frac{CGM-140}{140}{)}^{2})\right) \end{array}$$

During the evaluation, we had to use a median value when evaluating the tests, as the models performed very poorly for patients with lower body weight. Our tests showed that the Bump function performance was the best when the simulation was not stopped. The second best was the Cosine-type function. For the Bumps function, we obtained a TIR result of 68%. Also, the box plot shows very clearly the several outlier values. From the glucose curves, it turned out that there was a steady increase in the blood glucose curve. The CVGA plot shows that although the Bump function produced fewer Zone A control values, there were more Zone B and C values and fewer Zone D values.

In conclusion, CMG noise highly influences the performance of the models. However, a positive result is that these models avoid hypoglycemia, albeit at the expense of significant hyperglycemia. It also appears that the continuous functions perform much better than the original two-step reward function. The steady slope of the glucose curves suggests that one day of simulation is not enough, as this short period of time is not sufficient for the models to learn the transition between days. The best result was not far short of the expected 70% performance in terms of the TIR metric.

#### 3.2.3. Testing Differenct Avtivation Function and Hyperparameters

In our third experiment [56], we investigated how the changes in the hyperparameters affect the performance in terms of the CVGA and TIR metrics. The goal was to determine which changes in the hyperparameters are the most sensitive with respect to the control performance. To this end, we ran tests varying the number of neurons in the hidden layers between 64 and 512. In addition, we applied another test, where the number of neurons in the hidden layers was fixed (64 neurons), but the activation functions were modified simulation by simulation (Sigmoid, Relu, ELU, and No activation function). The environment was the same as in our second experiment with two characteristic inputs and one output, which was the amount of insulin.

Relu:
  (10) $$\begin{array}{c} f(x)=\max(0,x) \end{array}$$
Sigmoid:
  (11) $$\begin{array}{c} f\left(x\right)=\frac{1}{1+e^{-x}} \end{array}$$
ELU:
  (12) $$\begin{array}{c} f\left(x\right)=\left\{\begin{array}{cc} x & x\geq 0\\ \alpha (\exp(x)-1) & x<0 \end{array}\right. \end{array}$$

The training and test cases were the same as those used in the second test [53]. Also, the reward function was fixed (piecewise) to the one we originally used in our investigation, and we also used median values to summarize the results. Apparently, changing the number of neurons did not determine a large variation in the control ability of the neural network models, even if larger meshes performed marginally better than smaller meshes (the 512-neuron network achieved the best performance in the tests). Conversely, different activation functions resulted in significantly different performance outcomes of the controllers, with the ELU and the Sigmoid functions outperforming the others. For the ELU activation, a TIR metric above 70% was achieved. These results suggest that deeper meshes and ELU activation functions in the hidden layer should be used.

### 3.3. Virtual Patient Model

The basis of the environment to be controlled was provided by the Identifiable Virtual Patient model (IVP) [57]. We did not collect human data in our experiments. This model was extended with a model of sensor noise for our simulation environment. Our approach included a cohort of 10 specifically characterized patients, with additional parameters for night-time periods. This represents a total of 17 different virtual patient profiles. In total, we had 10 validated patient records to work with. However, as we also had daytime and night-time parameter sets for some patients, we had a total of 17 parameter sets. These parameter sets can be examined in Table 1.

The following equations describe the dynamics of a patient:

(13) $$\begin{array}{c} \begin{array}{cc} \dot{G}\left(t\right) & =-\left(\mathrm{GEZI}+I_{\mathrm{EFF}}\left(t\right)\right)\cdot G\left(t\right)+\mathrm{EGP}+R_{A}\left(t\right) \end{array} \end{array}$$

(14) $$\begin{array}{cc} {\dot{I}}_{\mathrm{EFF}}\left(t\right) & =-p_{2}\cdot I_{\mathrm{EFF}}\left(t\right)+p_{2}\cdot S_{I}\cdot I_{P}\left(t\right)\; \end{array}$$

(15) $$\begin{array}{cc} {\dot{I}}_{P}\left(t\right) & =-\frac{1}{\tau_{2}}IP\left(t\right)+\frac{1}{\tau_{2}}I_{SC}\left(t\right)\; \end{array}$$

(16) $$\begin{array}{cc} {\dot{I}}_{\mathrm{SC}}\left(t\right) & =-\frac{1}{\tau_{1}}I_{\mathrm{SC}}\left(t\right)+\frac{1}{\tau_{1}C_{I}}u\left(t\right)\; \end{array}$$

(17) $$\begin{array}{cc} R_{A}\left(t\right) & =\sum_{i}^{m}\frac{d_{i}}{V_{G}\cdot \tau_{D_{i}}^{2}}t_{i}\cdot e^{-\frac{t_{i}}{\tau_{D_{i}}}} \end{array}$$

Blood glucose concentration is symbolized as G(t) in units of milligrams per deciliter (mg/dL), and the effectiveness of insulin is denoted by $I_{EFF}\left(t\right)$ (min^−1^). Subcutaneous and plasma insulin concentrations are represented as $I_{\mathrm{SC}}\left(t\right)$ and $I_{P}\left(t\right)$, respectively, with both expressed in microunits per milliliter (μU/mL). The agent indirectly influences blood glucose level through the infusion of insulin. The $R_{A}$ encapsulates disturbances in the form of carbohydrate intake $d_{i}$ in grams.

The $\tau_{1}$ in minutes and $\tau_{2}$ in minutes are the timing parameters, and the rate constant $p_{2}$ (min^−1^) defines the absorption kinetics of insulin. Insulin clearance is $C_{I}$, in milliliters per minute (mL/min), while insulin sensitivity $S_{I}$ is expressed in milliliters per microunit per minute (mL/μU/min).

Endogenous glucose production, denoted by EGP, in units of milligrams per deciliter per minute (mg/dL/min), represents liver glucose output. Glucose effectiveness at zero insulin concentration (GEZI) describes insulin-independent glucose consumption. $V_{G}$ is the apparent glucose distribution volume in deciliters (dL). The time of meal absorption is described by the time constants $\tau_{D_{i}}$.

We extended the IVP model based on [58]. This augmentation consists of integrating into the IVP a CGM noise model, considering diverse factors such as temporal delay [59], sensor drift, additive sensor noise, and calibration imprecision. In order to avoid affecting the training process, we excluded the sensor drift from the model.

The temporal delay inherent to CGM measurements, a result of their interstitial measurement site, was incorporated into the extended model as an additional interstitial glucose compartment $I_{G}$. The sensor noise was modeled as an autoregressive process of second order with a stochastic white noise term, *w*, distributed as $N(0,\sigma^{2})$.

(18) $$\begin{array}{cc} \dot{IG}\left(t\right) & =-\frac{1}{\tau_{IG}}IG\left(t\right)+\frac{1}{\tau_{IG}}G\left(t\right), \end{array}$$

(19) $$\begin{array}{cc} \;v(t) & =\alpha_{1}v\left(t-T_{s}\right)+\alpha_{2}v\left(t-2T_{s}\right)+w\left(t\right),\; \end{array}$$

(20) $$\begin{array}{cc} CGM(t) & =IG(t)+v(t), \end{array}$$

### 3.4. Meal Generator

We utilized the meal generation schema of Wang et al. [60]. The algorithm generates six meals spread across the entire day, each having random carbohydrate content and timing.

- Assume that the patient’s body weight is known;
- Probability of meal appearance: p=[0.95,0.3,0.95,0.3,0.95,0.3];
- Meal time upper boundary: up=[9,10,14,16,20,23]·60;
- Meal time lower boundary: lo=[5,9,10,14,16,20]·60;
- Meal time: $\mu_{t}=\left[7,9.5,12,15,18,21.5\right]\cdot 60$;
- Meal time variance: $\sigma_{t}=\left[60,30,60,30,60,30\right]$;
- Amount of meal: $\mu_{a}=\left[0.7,0.15,1.1,0.15,1.25,0.15\right]\cdot BW$;
- Amount of meal variance: $\sigma_{a}=\mu_{a}\cdot 0.15$;
- Meal set: E=∅;
- For k∈[1,2,3,4,5,6] do
- Generate a random value for $p_{tmp}$ between 0–100;
- if $p_{tmp}\leq p\left[k\right]$
- Calculate the meal amount for kth timestamp: $e_{k}=Round\left(max\left(0,Normal\left(\mu_{a}\left[k\right],\sigma_{a}\left[k\right]\right)\right)\right)$
- Calculate the meal time for kth timestamp: $\zeta_{k}=Round\left(TruncNorm\left(\mu_{t}\left[k\right],\sigma_{t}\left[k\right],lb\left[k\right],op\left[k\right]\right)\right)$
- Make a union with the meal set: $E\cup \{e_{k},\zeta_{k}\}$;
- return E.

Since the mathematical model allows for carbohydrate intake, a method is needed to generate the carbohydrate input for the simulations. To enable the model to learn on more realistic simulations, it was important to implement this generator in a reinforcement learning environment. This solution is able to generate random carbohydrate inputs for all simulations based on a normal distribution. It takes the patient’s body weight into account and calculates carbohydrate intakes based on it, which is again realistic, since in real life patients determine their carbohydrate intake based on their body weight. In addition, it has 6 meals of which the main meal 95% is possible, and the side meal 30% is possible. This generator randomly generates the daily carbohydrate intake. When we used it for a simulation of several days, we generated as many days as we simulated, and we added the meals one after the other. When trainning the network, we always used random carbohydrate intake. We also regenerated the meal for each simulation.

### 3.5. Closed Loop in Terms of RL

**Figure 2:**
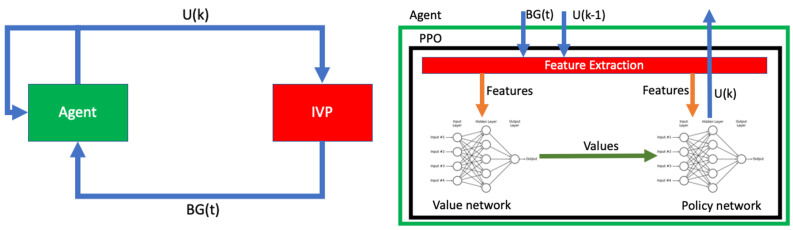
The Left side figure represents the block diagram of the reinforcement learning-based closed-loop algorithm. The right side figure details the control architecture of the system. BG(t) means blood glucose value measured at the t-th time point. U(k) insulin administered at time k.

In our approach, the virtual patient is the IVP model, while the RL agent behaves as a closed-loop controller administrating insulin (to the environment). The IVP model provides the dynamic output of blood glucose (BG) levels (noisy BG level) depending on an array of inputs including both carbohydrates and insulin. The RL agent computes the insulin dosage for the upcoming time step (5 min) by integrating prevailing BG levels and the accumulated history of administered insulin. The resulting closed-loop design is shown in Figure 2 and detailed in Equations (1)–(4).

### 3.6. Applied Neural Network

We used a Bump reward function to build our neural network. Similarly to our previous tests, our input feature vector consisted of two elements: the blood glucose level measured at the previous time and the amount of insulin administered at the previous time. The features were normalized to values between 0 and 1: for glucose, this was achieved by dividing the blood glucose level by 1000. For insulin, the action space in the environment was between [−1 and 1]; therefore, in the design of the feature vector, we added one to the current decision and then divided it by 2. The action space [−1,1] represented insulin administration values from 0 up to 25 U/h. When the network made a decision, we added 1 to that value, then multiplied that value by 25, and finally divided it by 2, which was the actual administered insulin value. Since our previous tests showed that the models perform better when they do not stop the simulation time if values exceed blood glucose limit values, we simulated the whole time period in any case. Since tests showed that a longer stimulation time is needed, we considered 10 days of simulation.

**Figure 3:**
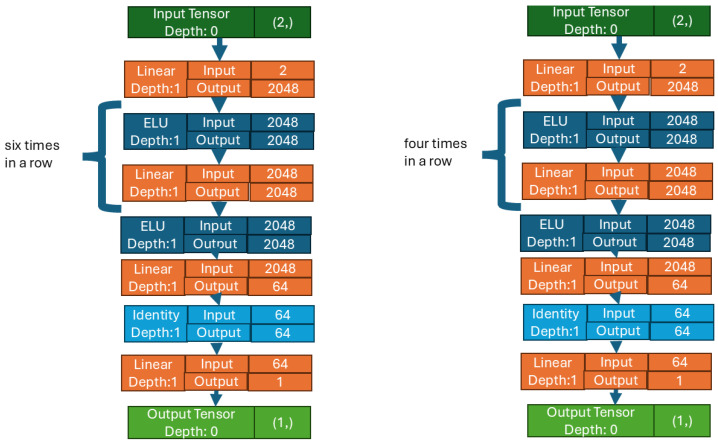
A graphical representation of the policy and value networks as depicted by Pytorch. The image on the left is the policy network, which is larger than the value network. This network learns the control strategy. On the right is the value network, which gives an estimate of how good the policy network was.

**Figure 4:**
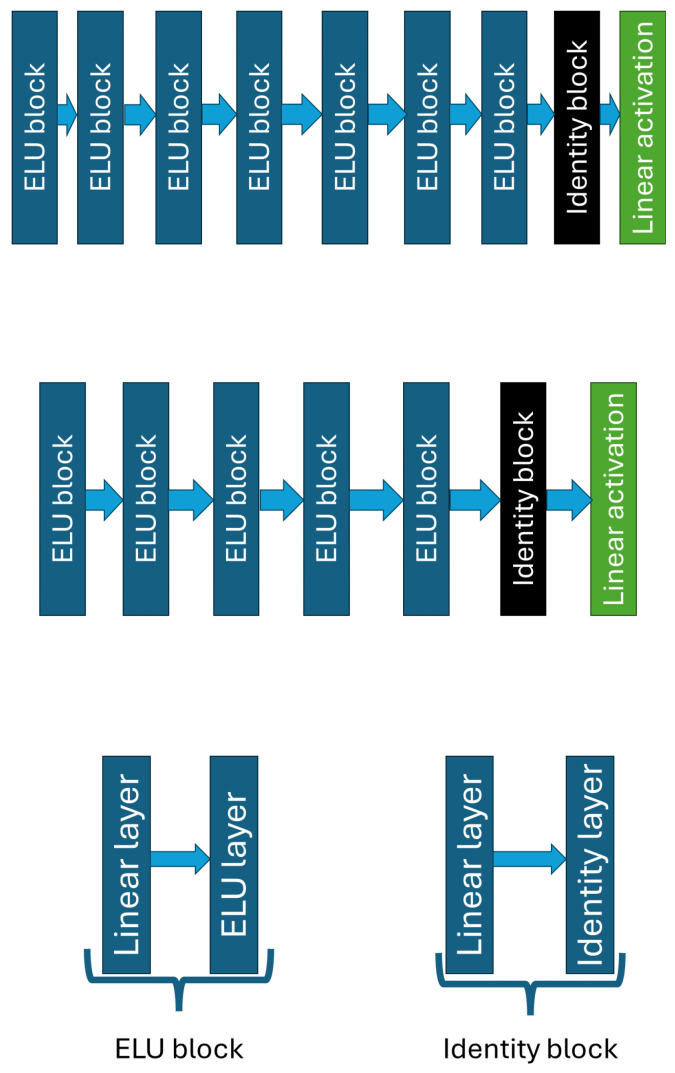
The **upper** figure shows the Policy network architecture. The **middle** figure shows the Value network architecture. The Block architectures are represented by the **bottom** figure.

Our current network shown in Figure 3 maintained the separation between value and policy as it was used in previous test. In this study, we applied large neural networks as blocks, consisting of Linear and ELU layers. The use of the ELU layer was justified by the good results obtained in the third test in Section 3.2.3, while the use of the Linear layer was applied to have continuous output. A variable number of these blocks appeared in the policy and value networks: while there were only five blocks in the value network, the policy network contained seven blocks. The value network was made smaller to allow the critical part of our model to learn faster, whereas the policy network was set deeper to give the actor more of a chance to learn the right strategy. Each block consists of 2048 neurons. The exceptions are the first block in both networks (the input layer of that block has 2 features) and the last block (which has an output of 64 values). Both networks had a terminal block consisting of an Identity layer and a Linear layer. The output of both networks is a single value: in the case of the value network, the value is a prediction of how good the given step was; for the policy network, the value, between −1 and 1, indicates the insulin quantity to be administered. Figure 4 shows the network architecture. The Linear layer was needed to obtain a linear combination in the output. In addition, in Pytorch 2.4.0 [61], where the Linear layer was used to vary the output number from the input. For other layers, the input number is the same as the output number. This output is then passed to the Identity layer.

All other hyperparameters Table 2 we used were the same as those used in the first test Section 3.2.1. We checked our agent’s reward value after each simulation and we considered the average reward achieved by the model during training, respectively. This test was performed for the last 100 simulations. If this average value increased from one iteration to the next. Then, we saved the weights of the model to archive the best model that performed the best. In this way, we were able to extract the best model, on average. We obtained these 100 simulations in our first experiments illustrated in Section 3.2.1 so that with this many simulations, we could obtain a good performance for insulin control. The first results were also published on this basis. So, we did not change this value. Also, we realized that the simulation time has a big impact on learning: we thus increased the simulation time, training the model over 50 million steps. We chose 50 million steps because we only had 5 million steps in the first test described in Section 3.2.1, while the second test also had 5 million steps, as described in Section 3.2.2. And in our third test, we also had 5 million steps Section 3.2.3. We observed that when we gave the agent more time to learn, it can achieve better results. Therefore, we decided that 50 million steps will be 10 times the learning time step so far. With this change, convergence (in the ELKH cloud [62]) took 2 or 3 weeks per patient.

### 3.7. Training Phase

Throughout the iterative training phase, a series of 10 consecutive day scenarios served as the test set to assess the evolving performance of the neural network. These scenarios incorporated randomized meal schemes interspersed between consecutive one-day periods, providing heterogeneous patterns for the networks to learn from. Different networks were trained for each subject, with constant patient parameters but varying timing and carbohydrate content of meals. The training was completed over 50 million iterative steps and was conducted on the ELKH research cloud.

### 3.8. Testing Phase

The phase of evaluation was carried over 13 different meal schemes—both random (10 schemes) and predefined (3 schemes) [53,56]. This makes the evaluation of performance possible on a comprehensive spectrum of challenges in terms of the meal intake. Furthermore, the objective here was to avoid overfitting; thus, the test cases were defined manually the as same a in the Section 3.2.3 testing scenarios. The virtual patient parameters were kept constant for both training and testing phases.

Among the 13 test scenarios, 10 replicated the methodology employed during the training phase, while three were manually designed to represent particular situations: the first was a scenario without any meal intake (0 g); and in the second, 12 g of carbohydrates were administered each hour. A single carbohydrate intake at 12 h is defined as the maximum amount of carbohydrate the generator can deliver plus 5g of carbohydrate.

### 3.9. Applied Metrics

We applied two different metrics for analyzing the result, which are the two most popular metrics for diabetic control:

- Control Variability Grid Analysis (CVGA);
- Time in Range (TIR).

**Figure 5:**
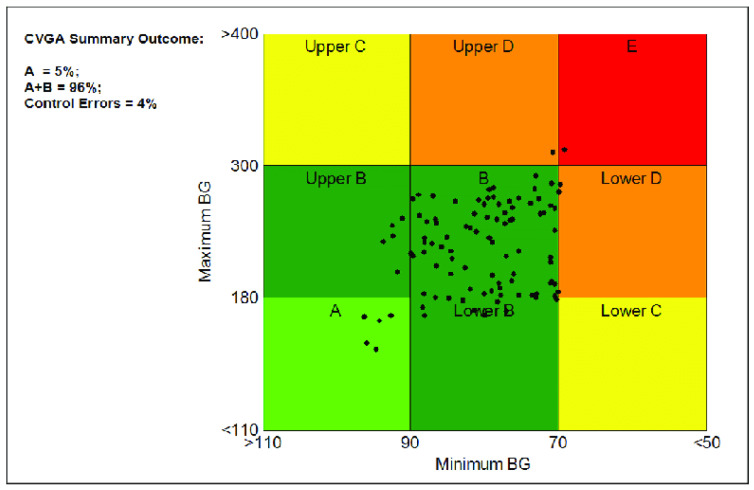
Control variability grid analysis (CVGA) metric.

Control Variability Grid Analysis (CVGA) Figure 5 control an algorithm’s performance. It provides insights into stability, responsiveness, and overall efficacy by assessing system behavior across various scenarios.

CVGA subjects control systems to different inputs or disturbances and measures responses, which are displayed on a grid. This visualization helps evaluate trade-offs between control objectives and optimizes system parameters.

Smith et al. offered an extensive overview of CVGA [63]. Johnson et al. demonstrated CVGA’s use in comparing control strategies [64]. Chen et al. showed the CVGA’s effectiveness in optimizing industrial process control systems [65].

The CVGA is essential for comprehensively assessing and optimizing control algorithms as the field evolves.

**Figure 6:**
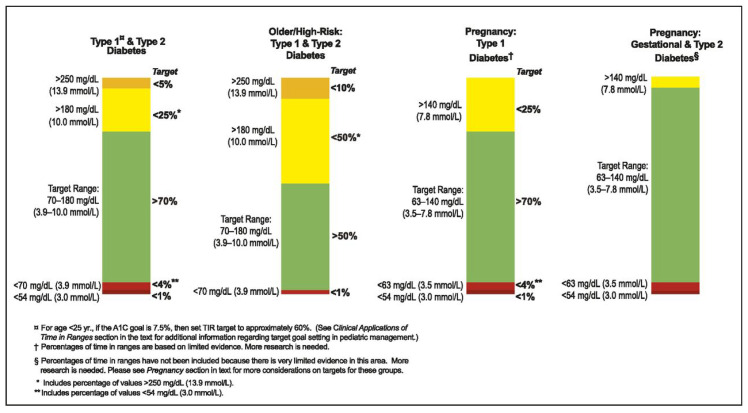
Time in range (TIR) metric.

The Time in Range (TIR) Figure 6 metric offers a comprehensive assessment of blood glucose control over time. Unlike traditional metrics focusing on extremes like hypoglycemia or hyperglycemia, the TIR quantifies the time spent within a target blood glucose range.

The TIR is expressed as the percentage of time blood glucose levels stay within a specified range, often 70–180 mg/dL reflecting optimal glycemic control. This range may vary based on individual factors and treatment goals.

The TIR captures the dynamic fluctuations in blood glucose levels, providing a holistic view of glycemic variability. It helps assess the effectiveness of treatment regimens, lifestyle changes, and therapy adjustments.

The International Consensus on Time in Range (ICTR) highlights the TIR’s importance in glycemic control. Battelino et al. showed the TIR’s association with reduced diabetes complications [66]. Beck et al. linked the TIR to quality of life in diabetes [67]. The ADA and ATTD provide guidelines on the TIR’s clinical use [68,69].

In summary, the TIR is a vital metric that offers a detailed evaluation of blood glucose control, supporting personalized diabetes management strategies.

## 4. Results

Table 3 lists the aggregated results in different glucose ranges. In the 70–180 mg/dL range the median value is high, 86% which surpasses previous results. It can be seen in 75% of the tests the method is able to achieve better than 44% TIR. The lowest value in this range is 18%, showing that there are still patients whose glucose control was not feasible with the current method. The outlier patients had low body weights in all cases. Overall, a TIR average greater than 70% was achieved, which is higher compared to our previous studies.

Based on the 180–250 mg/dL range, more than 75% of the patients remained under 180 mg/dL throughout the evaluation period. Similar observations can be seen to the >250 column as well, which indicates that it was only in certain scenarios where the blood glucose tended towards the larger values, but even those cases are within the permissible limit which limit is 5%.

The RMSE150 variable showed the root mean squared error from the 150 mg/dL glucose concentration; 50% of the data deviated from 150 by less than 37 mg/dL. Whereas if we also look at the upper quartile, 65 mg/dL is a reasonable result, as it can be said that a quarter of the test values range between 85 mg/dl and 215 mg/dL.

**Figure 7:**
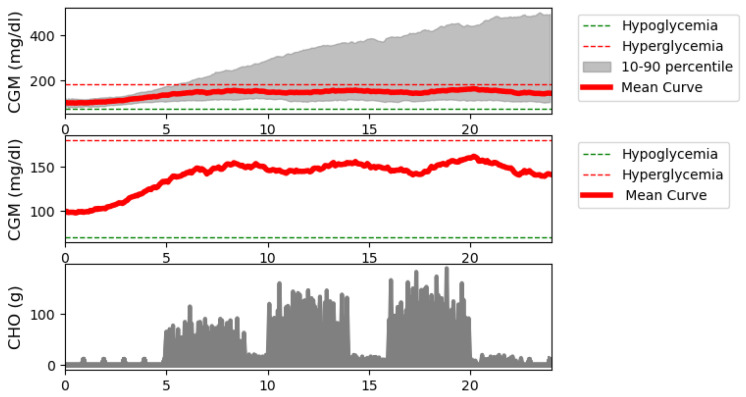
Blood glucose median curve with standard deviations and meal intakes for all the investigated days.

Figure 7 shows median and standard deviation of the 221 days simulated in the test. The median value was calculated for the blood glucose values of this simulation. This median value is also plotted on two sub-bars one, with only the median value and the other with the median value and values between the 10th and 90th percentiles. In addition, the carbohydrate intake is also shown. The median value is in the right range which shows satisfying performance. It is also a satifying result that the 10th percentile values are also in this range. However, there are patients whose simulated values go up to 400 mg/dL that indicating that the robustness of the system should be increased (this will be the focus of our further studies). These are mainly light-weight patients.

**Figure 8:**
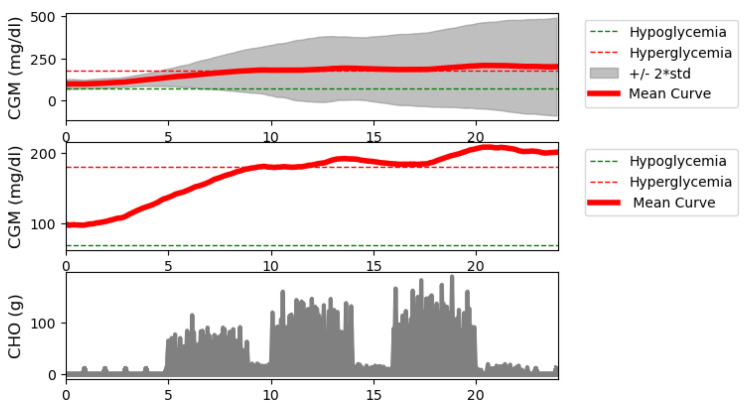
Mean blood glucose curve with standard deviation and meal intakes for all the investigated days.

The Figure 8 plot is similar to the Figure 7 plot. The difference is that the mean values are plotted for the data simulated during the test. Also, it is not the percentile difference that is shown, but the standard deviation +/−2. Notice that the mean also moved very much into the correct range until it left the range toward the end of the simulation. There is a noticeable steady increase in the blood glucose curve. This is due to the fact that there are simulations where the neural network model does not dose insulin, so there is a continuous increase in the blood glucose value. Since the average is sensitive to this, the simulation was in the high blood sugar range. The variance being large it is completely apparent. This also proves that the robustness of the RL controller is not satisfying in case of patients with lower weights. However, it is a satisfying result in that there is no high difference between median and mean blood glucose values.

**Figure 9:**
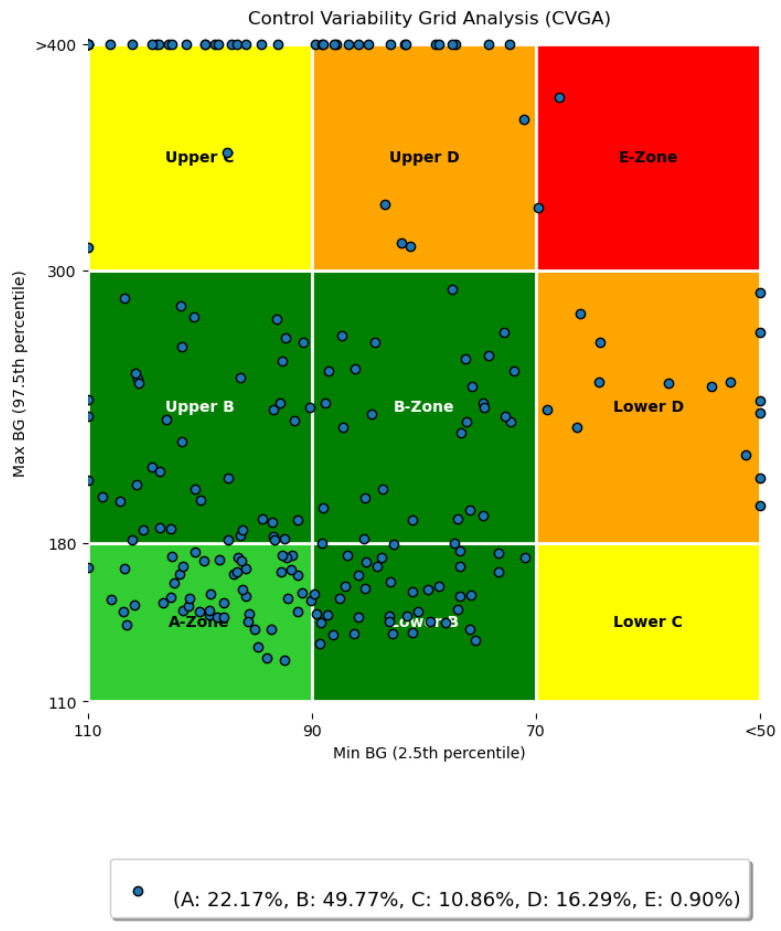
CVGA diagram for all tested days.

In Figure 9 the CVGA is given. A large portion of the samples lie in the Zone A and Zone B. Compared to around 1% Zone A in our previous study, Section 3.2.2 and Section 3.2.3, the method now reached 22% fir Zone A, which is a significant improvement. Similarly, the number of Zone B occurrences also increased compared to our preliminary results in Section 3.2.2 and Section 3.2.3 from around 30% to almost 50%. Zone C occurrences remained at the same level as before. Samples in Zone Upper D were greatly reduced, approximately halved. Also, zone E samples were minimized. The spikes can be seen in both the high blood sugar level section and the low blood sugar level section. At each of the two extremes (above 400 mg/dL and lower than 50 mg/dL alongside the axis) produced by the virtual patients with lower weights (lower than cca. 65 kg). In our further study, we will investigate this phenomenon. In general, the model mostly avoids hypoglycemia and hyperglycemia, however, in some cases, both hypo- and hyper glycemia occur.

## 5. Discussion

After illustrating our results, we will now look in more depth at all the results we have achieved. Let us start with the Table 3, where it was already mentioned that the aggregate results are shown for all simulated days. For the value <50, notice that half of the simulated days have a value of 0, since the value of 50% has a value of 0. For the value 75% there is a value of 11. This means that 25% of the simulated days fell into the very hypoglycemic zone. There were 55 days where the blood sugar went below 50 mg/dL during the simulation. What is worse is that in the max row, you can see a value of 73, which shows that there are patients for whom our model absolutely cannot work. These cases occur in patients with a lighter body weight. The model suddenly injects a large amount of insulin into the patient, causing a very low blood glucose level, and the patient is unable to get back into the correct range. This can be avoided by not administering insulin when blood glucose levels are already very low. Looking at the next column, which is 50–70, we see that at least half of the simulations are below 9. However, a quarter of the simulated days have no value in this zone. In row, 75% the value doubled. So for many patients, blood glucose levels move in this zone during the simulation. According to the TIR metrics, you should not spend more than 5 min in this zone. However, it can be said that if the value does not go below 50 mg/dL, it is not a serious problem. Now the most important column is the TIR metric value. It is very good that more than 50% of the simulated days satisfy the TIR metric with a value greater than 70 in column 70–180. In fact, the 75% row shows that a quarter of the simulated days are 100% in this zone. The average value is also greater than 70%, which was not seen in our previous tests. Furthermore, 25% should show that a quarter of the simulated days can only achieve less than 40%. The next two columns will be discussed together. To the extent that our previous studies were large, the values of these columns are minimal here to show that the models do not learn by not injecting insulin. However, it can be seen that you can have days where there were high blood glucose values. These also tend to happen in lightweight patients, Because the model reacts too late to sudden spikes in blood sugar when there is carbohydrate intake.

Next are Figure 7 and Figure 8, where the simulation results for all days are shown, including the mean and median curves calculated from these days. For the mean, +/−2 standard deviations, and for the median, the value between the 10th and 90th percentiles are plotted. In addition, we examined the carbohydrate intakes. Starting with the carbohydrate intakes, the graphs clearly show that the three main meals appear in larger sizes. And the shifts are also clearly visible. This would prove that very random days were used to perform the tests. In the case of the median curve, it is clearly visible that our model structure picture of staying in the TIR zone even at the end of the day did not allow us to leave this zone. However, it can be seen that at the beginning. the models allowed the blood glucose level to rise and then stabilize around 150 mg/dL. Examining the picture on which the percentiles are also shown, we can say that the TIR range is not allowed to be exceeded even for the 10th percentile. The bottom of the variance is still within the range. However, for the 90th percentile, we can see that the upper range reached 400 mg/dL, which is very high. So, our model structure still does not handle hyperglycaemia in the best way. On the other hand, it handles hypoglycaemia very well. The vast majority of tests are not even in the hypoglycemic range. What you will notice is that there is a steady increase in blood glucose. This proves that 10 days of study is too long. The following should reduce the simulation time used for teaching. Let us turn to the figure showing the average. Unlike the median value, the average value here is out of the TIR range by the end of the day. And you can see a steadily increasing slope. This proves our previous claim that the learning simulation time is too long. However, the average curve did not exceed 200 mg/dL. Looking at the scatter plot, it can be seen that the maximum value can reach 500. So, we can say that there are patient cases for which our solution does not work absolutely well. This is true for patients with light weight. Because the model in their case reacts late or not at all—because for these patients, if the insulin is administered before the time of administration, it causes a very big change in their blood glucose. Next is the CVGA Figure 9 shows all the test days. It can be seen that in one A aand in Zone B which is the most important in terms of the goodness of control our value at 72% which means almost three-quarters of the days that we can solve with good control. It can also be seen that the days are shifted to the upper range for bad controls, as there being much more for Upper C and Upper D than for Lower C and Lower D. For our Zone E control, which is the completely bad control, we have hardly any value of days 1% is there. Looking at the graph, this means specifically one day and two are on the borderline. So we can say for the vast majority of days our solution works well. However, in the cases where it does not work well, most of the time the control moves toward the high blood glucose side. To a lesser extent, it moves to the low blood sugar.

## 6. Conclusions

We developed a reinforcement learning-based closed-loop controller that is able to bring the median TIR metric value towards 80%. In addition, the number of Zone A and Zone B samples in the CVGA analysis was substantially increased compared to our previous studies. This new approach is able to generally stabilize the glycemic curves, greatly improving the TIR values at the cost of a slight increase in the TBR values. Hypoglycemias appeared to occur in patients weighing less than 65 kg: finding methods to effectively and safely control patients with lower body weight is a goal in future studies, since apparently the current model does not generalize well in their case. Simulation time is also of concern and should be reduced in further studies. Other issues to be tackled concern the policy algorithm and the fact that during tests the PPO model could fail by getting stuck in a local minimum. In addition, learning with a much larger feature vector should be evaluated, since this could solve the problem of administering excessive doses of insulin at once.

## Figures and Tables

**Table 1:** Patient parameter sets, in cases where the VG and BW parameters are the same, are the same patient.

BW	GEZI	EGP	CI	SI	tau1	tau2	p2	VG
89	$3.87\times 10^{-8}$	1.4	2010	$4.93\times 10^{-4}$	49	47	$1.06\times 10^{-2}$	253
89	$2.20\times 10^{-3}$	1.33	2010	$8.11\times 10^{-4}$	49	47	$1.06\times 10^{-2}$	253
63	$4.38\times 10^{-3}$	0.6	1281	$9.64\times 10^{-5}$	41	10	$1.16\times 10^{-2}$	261
65	$3.50\times 10^{-3}$	0.856	909	$1.70\times 10^{-4}$	71	70	$2.33\times 10^{-2}$	199
65	$1.64\times 10^{-3}$	1.07	909	$4.63\times 10^{-4}$	71	70	$2.33\times 10^{-2}$	199
116	$7.58\times 10^{-8}$	2.59	1813	$3.77\times 10^{-4}$	91	70	$8.14\times 10^{-3}$	337
116	$1.64\times 10^{-5}$	0.98	1813	$3.77\times 10^{-4}$	91	70	$8.14\times 10^{-3}$	337
64	$4.33\times 10^{-3}$	0.6	1535	$2.05\times 10^{-4}$	46	46	$9.63\times 10^{-3}$	188
51	$1.01\times 10^{-3}$	0.603	588	$4.12\times 10^{-4}$	68	30	$9.15\times 10^{-3}$	104
77	$2.30\times 10^{-3}$	1.11	1806	$8.16\times 10^{-4}$	60	60	$1.01\times 10^{-2}$	263
65	$1.00\times 10^{-8}$	1.3	540	$3.68\times 10^{-4}$	95	37	$1.03\times 10^{-2}$	137
100	$6.39\times 10^{-3}$	1.27	875	$2.56\times 10^{-4}$	131	21	$1.03\times 10^{-2}$	193
64	$1.04\times 10^{-3}$	0.611	1309	$6.03\times 10^{-4}$	53	53	$1.02\times 10^{-2}$	204
51	$3.79\times 10^{-3}$	0.603	588	$9.48\times 10^{-4}$	68	30	$9.15\times 10^{-3}$	104
65	$1.00\times 10^{-8}$	0.601	540	$5.40\times 10^{-4}$	95	37	$1.03\times 10^{-2}$	137
100	$6.39\times 10^{-3}$	3.45	875	$6.89\times 10^{-4}$	131	21	$1.03\times 10^{-2}$	193
64	$1.04\times 10^{-3}$	0.611	1309	$1.73\times 10^{-3}$	53	53	$1.02\times 10^{-2}$	204

**Table 2:** Hyperparameters of the agent.

Hyperparameter	Value	Description
learning rate	0.0003	
number of steps	288	The number of steps to run for each environment per update
batch size	64	
gamma	0.99	
lambda	0.95	Factor for trade-off of bias vs variance for Generalized Advantage Estimator.
clip range	0.2	Clipping parameter—it is a function of the current progress remaining.
entropy coefficient	0	Entropy coefficient for the loss calculation.
value function coefficient	0.5	Value function coefficient for the loss calculation.
max gradient norm	0.5	The maximum value for the gradient clipping.
use SDE	False	Whether to use generalized State Dependent Exploration (gSDE) instead of action noise exploration.
target KL	None	Limit the KL divergence between updates, because the clipping is not enough to prevent large.

**Table 3:** Aggregated table for all investigated simulation days, including mean, maximum, minimum, standard deviation, and quarterlies values in terms of TIR zones.

	<50	50–70	70–180	180–250	>250	RMSE90	RMSE150
mean	11.925	14.013	73.261	0.512	0.289	94.777	69.905
std	21.647	17.288	28.350	1.482	1.466	79.771	67.590
min	0.000	0.000	18.056	0.000	0.000	20.016	17.106
25%	0.000	0.000	44.792	0.000	0.000	43.382	31.384
50%	0.000	8.333	86.806	0.000	0.000	61.408	37.472
75%	11.458	22.222	100.000	0.000	0.000	109.674	65.366
max	73.264	73.264	100.000	9.375	11.458	360.283	309.865

## Data Availability

The original contributions presented in the study are included in the article, further inquiries can be directed to the corresponding author.

## Abbreviations

The following abbreviations are used in this manuscript:

DM	Diabetes mellitus
T1DM	Type 1 Diabetes Mellitus
T2DM	Type 2 Diabetes Mellitus
AP	Artificial pancreas
CGMS	Continuous Glycemia Monitoring System
PID	Proportional–Integral–Derivative
MPCs	Model Predictive Controllers
RL	Reinforcement Learning
IVP	Identifiable Virtual Patient model
GEZI	Glucose effectiveness at Zero Insulin concentration
EGP	Endogenous Glucose Production
PPO	Proximal Policy Optimization
BG	Blood Glucose Levels
TIR	Time In Range
CVGA	Continuous Variability Grid Analysis
